# Copper Ions Induce DNA Sequence Variation in Zygotic Embryo Culture-Derived Barley Regenerants

**DOI:** 10.3389/fpls.2020.614837

**Published:** 2021-02-04

**Authors:** Renata Orłowska, Janusz Zimny, Piotr T. Bednarek

**Affiliations:** Plant Breeding and Acclimatization Institute–National Research Institute, Błonie, Poland

**Keywords:** barley, copper, silver, metAFLP, somatic embryogenesis, mediation analysis

## Abstract

*In vitro* tissue culture could be exploited to study cellular mechanisms that induce sequence variation. Altering the metal ion composition of tissue culture medium affects biochemical pathways involved in tissue culture-induced variation. Copper ions are involved in the mitochondrial respiratory chain and Yang cycle. Copper ions may participate in oxidative mutations, which may contribute to DNA sequence variation. Silver ions compete with copper ions to bind to the complex IV subunit of the respiratory chain, thus affecting the Yang cycle and DNA methylation. The mechanisms underlying somaclonal variation are unknown. In this study, we evaluated embryo-derived barley regenerants obtained from a single double-haploid plant *via* embryo culture under varying copper and silver ion concentrations and different durations of *in vitro* culture. Morphological variation among regenerants and the donor plant was not evaluated. Methylation-sensitive Amplified Fragment Length Polymorphism analysis of DNA samples showed DNA methylation pattern variation in CG and CHG (H = A, C, or T) sequence contexts. Furthermore, modification of *in vitro* culture conditions explained DNA sequence variation, demethylation, and *de novo* methylation in the CHG context, as indicated by analysis of variance. Linear regression indicated that DNA sequence variation was related to *de novo* DNA methylation in the CHG context. Mediation analysis showed the role of copper ions as a mediator of sequence variation in the CHG context. No other contexts showed a significant sequence variation in mediation analysis. Silver ions did not act as a mediator between any methylation contexts and sequence variation. Thus, incorporating copper ions in the induction medium should be treated with caution.

## Introduction

Barley (*Hordeum vulgare* L.) is a prominent grain used for human consumption, animal feeding, brewing, and renewable energy production (Tiidema and Truve, 2004; Schulze, 2007). Because of its importance as a food crop, barley receives much attention with regard to *in vitro* tissue culture and plant regeneration through somatic embryogenesis (Sharma et al., 2005). The improvement of agronomic traits, such as quality and disease resistance, and the development of gene modification techniques require efficient protocols for plant regeneration *via in vitro* tissue culture. However, the efficiency of plant regeneration through somatic embryogenesis varies with the plant genotype (Sharma et al., 2005). In barley, somatic embryogenesis has been performed using various explants, including mature and immature zygotic embryos (Lupotto, 1984; Bregitzer et al., 2000; Tiidema and Truve, 2004; Sharma et al., 2005; Ganeshan et al., 2006). The resultant regenerants showed phenotypic (Ruíz et al., 1992) and genotypic variation (Temel et al., 2014). Furthermore, barley regenerants derived through embryo culture also exhibited genetic and epigenetic variability (Bednarek et al., 2007).

Tissue culture-induced variation (TCIV) frequently arises during *in vitro* plant regeneration (Fiuk et al., 2010; Mikula et al., 2011a; Machczyńska et al., 2014a). The process of plant regeneration requires cell reprogramming (Maki, 2002), which can result in genetic (sequence) and epigenetic (DNA demethylation and *de novo* methylation) changes (Miyao et al., 2011; Weckx et al., 2019; Orłowska and Bednarek, 2020). DNA methylation changes can further lead to sequence variation because of the activation of mobile elements (Orłowska et al., 2016) or perturbations in DNA repair systems (Wang et al., 2013) under *in vitro* tissue culture conditions. However, other reasons such as reactive oxygen species (ROS), plant growth regulators, number of subculture for TCIV cannot be excluded (Bairu et al., 2011; Krishna et al., 2016; Bednarek and Orłowska, 2020b).

Plant regeneration *via in vitro* tissue culture involves the use of varying concentrations of diverse ingredients, such as copper (Cu^2+^) and silver (Ag^+^) ions (Orłowska and Bednarek, 2020). Copper ions participate in the electron transport chain (Pádua et al., 2010; Tan et al., 2010; Ravet and Pilon, 2013; Printz et al., 2016) and cell wall metabolism (Printz et al., 2016) and act as cofactors that facilitate ethylene receptors to bind to ethylene (Hirayama and Alonso, 2000; Light et al., 2016). Copper ions also protect cells against oxidative damage (Szöllösi, 2014) and contribute to the production of hydroxyl radicals *via* the Haber–Weiss cycle (Ravet and Pilon, 2013) and to a wide range of biochemical pathways affecting DNA methylation (Cong et al., 2019; Puchkova et al., 2019). At sublethal concentrations, copper ions influence the Krebs cycle (Lauer et al., 2012).

Silver ions affect the number of green regenerants obtained *via* anther culture (Jha et al., 2007); induce embryogenic callus formation (Vain et al., 1989; Songstad et al., 1991); stimulate shoot induction, shoot formation (Purnhauser et al., 1987), and *in vitro* rooting (Wu et al., 2006; Jha et al., 2007; Würschum et al., 2015); inhibit callus necrosis (Wu et al., 2006); and affect organogenesis (Paladi et al., 2017). Silver ions can also compete with copper ions to bind to the mitochondrial complex IV (Beyer, 1976; Costa et al., 2010) and to function as a cofactor of ethylene receptors for ethylene binding (Light et al., 2016). Moreover, silver ions induce sequence variation because of disturbances in DNA methylation processes during barley anther culture (Bednarek and Orłowska, 2020a).

Although some pieces of evidence support the role of copper and silver ions in barley anther culture, limited information is available on the effect of these ions on embryo-derived regenerants; an exception is our recent study on barley, where we optimized the culture conditions for maximizing the number of regenerants (Orłowska et al., 2020). It is not evident whether the L-methionine cycle is involved in zygotic embryo culture-induced variation. Since plant regeneration *via* zygotic embryo culture avoids chromosome doubling, the reestablishment of DNA methylation patterns *via* DNA replication might be facilitated (Piccolo and Fisher, 2014), limiting (epi)genetic pattern changes. However, the role of epigenetic processes, such as base excision repair (BER) (Drohat and Coey, 2016), nucleotide excision (NER) (Zhang et al., 2019), mismatch repair (MMR) (Marinus, 2012), 5-hydroxymethylcytosine (5-mC) oxidation (Globisch et al., 2010), and other modifications (Ito et al., 2011), in DNA methylation cannot be excluded (Liu and Lang, 2020). Active DNA demethylation can lead to C → T mutations, and 5-mC oxidation can also contribute to sequence variation (Nabel et al., 2012).

A key player in *de novo* DNA methylation processes, as well as in the methylation of other cellular compounds (Giovanelli et al., 1985), is S-adenosyl-L-methionine (SAM) (Cantoni, 1952), a product of the Yang cycle (Mäkinen and De, 2019). The Yang cycle is linked to the mitochondrial respiratory chain (Tripodi et al., 2018) *via* adenosine triphosphate (ATP), which is required for the synthesis of SAM (Markham and Pajares, 2009). Perturbations in the respiratory chain (Pu et al., 2015) affect the L-methionine cycle (Kanyuka and Rudd, 2019). The lack or excess of SAM alkylating agent at the DNA methylation level leads to mobile element activation (Yilmaz et al., 2014) and/or mutations (Nabel et al., 2012). When copper and silver ions are included in tissue culture media, the mitochondrial respiratory chain enzyme, cytochrome c oxidase (Horn and Barrientos, 2008), may cause an imbalance, influencing the L-methionine cycle and altering relationships between DNA methylation and sequence variation. Additionally, copper and silver ions can induce abiotic stress (Speranza et al., 2013; Nawrot-Chorabik, 2017), leading to TCIV (Krishna et al., 2016).

Changes in DNA methylation patterns have been evaluated using Diversity Arrays Technology Sequencing Methylation Analysis (DArTseqMet) (Pereira et al., 2020), semiquantitative methylation-sensitive amplification polymorphism (MSAP) (Bednarek et al., 2017), methylRAD (Wang et al., 2015), methyl-seq (Brunner et al., 2009), PacBio (Rhoads and Au, 2015), and a variant of the MSAP approach (Baranek et al., 2016). Sequence variation and DNA methylation changes affecting distinct DNA sequence contexts (symmetric and asymmetric) could be analyzed using the methylation-sensitive Amplified Fragment Length Polymorphism (metAFLP) method (Orłowska and Bednarek, 2020). This method takes advantage of the properties of *Acc*65I and *Kpn*I isoschizomers, which recognize the same restriction site but differ in their sensitivity toward the DNA methylation pattern at the restriction site (Bednarek et al., 2007; Mikula et al., 2011b; Coronel et al., 2018). We recently used metAFLP to analyze DNA methylation patterns in barley regenerants derived *via* anther culture (Bednarek and Orłowska, 2020a, c; Bednarek et al., 2020).

We assume that copper and possibly silver ions added to the *in vitro* tissue culture medium influence the mitochondrial respiratory chain, thus affecting ATP production and influencing the Yang cycle. As the production of S-adenosyl L-methionine is concerned, the DNA methylation processes may be changed. Furthermore, copper ions participate in the oxidation of methylated cytosines, thus inducing mutations; although this has been verified in anther culture-derived barley regenerants, no information is available on embryo culture-derived regenerants. Understanding the processes that lead to TCIV in regenerants obtained *via* anther and embryo tissue culture may reveal putative differences and similarities between the two approaches.

This study aims to assess the influence of copper and silver ions on the relationship between DNA methylation sequence contexts (CG and CHG, where H = A, C, or T) and sequence variation during the immature zygotic embryo culture of barley.

## Materials and Methods

### Tissue Culture and Plant Regeneration

Spring barley line NAD2 (Poznan Plant Breeders LTD., Nagradowice, Poland) was used to regenerate double-haploid (DH) plants (Orłowska and Bednarek, 2020) *via* spontaneous doubling (Segui-Simarro and Nuez, 2008), followed by the evaluation of generative progeny called donor plants (Supplementary Figure S1).

Twenty-four donor plants were cultivated in a growth chamber under controlled conditions (16-h light/8-h dark photoperiod, 16°C day/12°C night temperature, and 190 μE m^–2^ s^–1^ light intensity). Spikes with immature caryopses were collected 12–16 days after self-pollination. The caryopses were sterilized by soaking in 70% ethanol for 1 min, followed by stirring in 10% sodium hypochlorite for 20 min. The sterilized caryopses were rinsed several times with sterile water (Makowska et al., 2017) and used for immature zygotic embryo extraction. The extracted immature zygotic embryos were placed in Petri dishes (60 × 15 mm) containing solid N6L medium comprising macro- and micronutrients (Chu, 1978) and supplemented with 2 mg l^–1^ 2,4-dichlorophenoxyacetic acid (2,4-D), 0.5 mg l^–1^ 6-naphthalene acetic acid (NAA), and 0.5 mg l^–1^ kinetin. These embryos were incubated at 26°C under a 16 h light/8 h dark photoperiod to induce callus and somatic embryo formation. Nine different types of induction media (based on N6L), each supplemented with varying concentrations of CuSO_4_ and AgNO_3_, were tested (trials M1–M9) (Table 1). Furthermore, different durations of incubation on induction medium were tested (i.e., starting from planting the immature embryo on induction medium to when calli, first somatic embryos, or embryo-like structures were placed on regeneration medium) (Table 1). In total, 2,150 immature embryos were placed on the induction media throughout the experiment. Each variant of the experiment (M1–M9) had about 235 embryos. Primary explant-derived calli, somatic embryos, and embryo-like structures were observed several days after incubation on induction medium. Calli, embryo-like structures, and somatic embryos were transferred from the induction medium to the K4NB regeneration medium (Kumlehn et al., 2006) supplemented with 0.225 mg l^–1^ 6-benzylaminopurine (BAP) at 21, 28, and 35 days, respectively. Calli and embryo-like structures were incubated on regeneration medium at 26°C, 16 h light/8 h dark photoperiod, and 50 μE m^–2^ s^–1^ light intensity. Green regenerated plantlets were transferred to 100 ml glass flasks containing N6I rooting medium (Chu, 1978) supplemented with 2 mg l^–1^ indole-3-acetic acid (IAA). Subsequently, the regenerants were planted in a soil:sand mixture (3:1) and grown to maturity in a greenhouse under the same day/night regime as that described above for donor plants. Finally, 45 regenerants that originated from a single donor plant and equally representing each trial (five regenerants per trial) were used for further experiment. One donor plant and all regenerants derived from this donor constituted one set.

**TABLE 1:** Characteristics of 45 barley regenerants derived *via* somatic embryogenesis under varying concentrations of copper and silver ions in the induction medium and different tissue culture durations (Time).

Components added to the induction medium		Length of induction	Trial^†^	Quantitative characteristics evaluated by metAFLP (%)§				
Cu^2+^ (μM)	Ag^+^ (μM)	Time (days)		CG_DMV	CHG_DMV	CG_DNMV	CHG_DNMV	SV
0.1	0	21	M1	0.495557	0.480178	0.495557	0.960355	1.919313
0.1	0	21	M1	0.495557	0.479323	0.495557	0.958647	1.918407
0.1	0	21	M1	0.487013	0.479323	0.974026	0.958647	1.917198
0.1	0	21	M1	0.487013	0.480178	0.974026	0.960355	1.918029
0.1	0	21	M1	0.487013	0.479217	0.487013	0.958433	2.395460
0.1	10	28	M2	0.491285	0.479133	0.000000	0.958267	3.354650
0.1	10	28	M2	0.487013	0.479964	0.487013	0.959928	2.396498
0.1	10	28	M2	0.484877	0.479133	0.969754	0.958267	3.353934
0.1	10	28	M2	0.512645	0.479217	0.000000	0.958433	2.398008
0.1	10	28	M2	0.489861	0.480178	0.489861	0.960355	2.398575
0.1	60	35	M3	0.489861	0.479323	0.979722	0.958647	1.918029
0.1	60	35	M3	0.489861	0.479217	0.489861	0.958433	2.876564
0.1	60	35	M3	0.495557	0.479323	0.495557	0.958647	1.918407
0.1	60	35	M3	0.491285	0.480178	0.491285	0.960355	1.918407
0.1	60	35	M3	0.491285	0.479217	0.491285	0.958433	2.396498
5	60	28	M4	0.487013	0.958267	0.487013	0.958267	2.875085
5	60	28	M4	0.504101	0.959928	0.000000	0.959928	1.918407
5	60	28	M4	0.491285	0.958433	0.491285	0.958433	1.917198
5	60	28	M4	0.491285	0.959928	0.491285	0.959928	1.918029
5	60	28	M4	0.487013	0.958433	0.487013	0.958433	1.916368
5	0	35	M5	0.504101	0.959928	0.000000	0.959928	1.918407
5	0	35	M5	0.487013	0.958433	0.974026	0.958433	1.916943
5	0	35	M5	0.491285	0.958267	0.000000	0.958267	2.875414
5	0	35	M5	0.487013	0.958433	0.487013	0.958433	1.916368
5	0	35	M5	0.487013	0.958433	0.487013	0.958433	1.916368
5	10	21	M6	0.484165	0.958433	0.968330	0.958433	1.916176
5	10	21	M6	0.484165	0.958267	0.968330	0.958267	2.395015
5	10	21	M6	0.487013	0.958134	0.487013	0.958134	2.874017
5	10	21	M6	0.487013	0.958134	0.487013	0.958134	2.874017
5	10	21	M6	0.487013	0.95933	0.487013	0.95933	2.875085
10	10	35	M7	0.484165	0.958025	0.968330	0.958025	3.352553
10	10	35	M7	0.484877	0.958025	0.969754	0.958025	3.832442
10	10	35	M7	0.491285	0.958025	0.491285	0.958025	4.790227
10	10	35	M7	0.487013	0.958025	0.487013	0.958025	3.832735
10	10	35	M7	0.504101	0.959113	0.000000	0.959113	3.355512
10	60	21	M8	0.489354	0.964543	0.978709	1.446815	3.371571
10	60	21	M8	0.493647	0.963542	0.493647	1.445313	3.370862
10	60	21	M8	0.489354	0.963542	0.978709	1.445313	3.370540
10	60	21	M8	0.492216	0.963388	0.984432	1.445081	3.852831
10	60	21	M8	0.493647	0.963542	0.493647	1.445313	3.370862
10	0	28	M9	0.492216	0.963542	0.984432	1.445313	3.371571
10	0	28	M9	0.497940	0.963542	0.49794	1.445313	3.371958
10	0	28	M9	0.492216	0.963542	0.984432	1.445313	3.371571
10	0	28	M9	0.489354	0.963542	0.978709	1.445313	3.370540
10	0	28	M9	0.489354	0.963542	0.978709	1.445313	3.370540
			Mean	0.490954	0.800030	0.597614	1.066947	2.719493
			SD	0.005934	0.22919	0.326757	0.204601	0.746831

*^†^Trials (M1–M9) indicate conditions used to evaluate regenerants; SD, standard deviation.§ metAFLP, methylation-sensitive Amplified Fragment Length Polymorphism; CG_DMV and CHG_DMV, DNA demethylation of the CG sequence context; CG_DNMV and CHG_DNMV, de novo DNA methylation of the CHG sequence context; SV, sequence variation.*

### DNA Extraction

Genomic DNA was extracted from the fresh leaves of 45 regenerants and a donor plant using the Plant DNeasy MiniPrep Kit (Qiagen, Hilden, Germany). DNA purity and concentration were tested using a spectrophotometer at wavelengths of 260 and 280 nm, whereas DNA integrity was verified by 1.2% agarose gel electrophoresis and ethidium bromide staining.

### DNA Analysis by Methylation-Sensitive Amplified Fragment Length Polymorphism

The metAFLP analysis was performed as described previously (Bednarek et al., 2007), with minor modifications (Machczyńska et al., 2014b). Briefly, each DNA sample was separately digested with *Acc*65I/*Mse*I and *Kpn*I*/Mse*I following adaptor ligation, pre-selective amplification, and selective amplification steps. Eight selective primer pairs were used for PCR amplification (Supplementary Table S1). The P^32^-labeled PCR products were separated on polyacrylamide gels, and the gels were exposed to X-ray films. The DNA banding patterns were converted into a binary contraposed matrix representing profiles of the *Acc*65I/*Mse*I- and *Kpn*I*/Mse*I-derived platforms. The banding patterns were assigned to DNA sequence and DNA methylation change events and quantified, delivering information on sequence variation (SV), *de novo* DNA methylation (DNMV), and DNA demethylation (DMV). Furthermore, the metAFLP characteristics of varying DNA methylation contexts (CHH, CHG, and CG) were calculated (Orłowska and Bednarek, 2020).

### Statistical Analysis

Analysis of variance (ANOVA) was conducted in XlStat (Addinsoft, 2020). Excel add-in, whereas correlation and linear regression were performed in SPSS v 25 (IBM Corp, 2017). Mediation analysis was carried out in SPSS v 25 (IBM Corp, 2017) using PROCESS MACRO (Hayes, 2018). The Goodman test was calculated based on *t* statistics evaluated for paths *a* and *b* of mediation analysis using a calculator for the Sobel test by K. J. Preacher^1^. Variance accounted for the mediation was expressed as the *B* coefficient of indirect and total mediation effects.

## Results

Although many embryos from 24 donor plants were plated, only in the case of one donor plant we succeeded in regeneration of plants that fulfilled requirements of our experimental design [a single donor plant and its regenerants representing all experimental conditions (trials M1–M9) and had at least five regenerants per trial]. All anther culture-derived and acclimatized donor plants were morphologically uniform in terms of leaf shape and color, plant height, spike number, and seed number (Supplementary Figure S2) and exhibited no differences compared with the source plant materials. *In vitro* immature zygotic embryo culture yielded as many as 45 regenerants from a single donor plant. Five regenerants were tested in each trail (M1–M9). All regenerated plants were morphologically identical to the donor plant.

**FIGURE 1:**
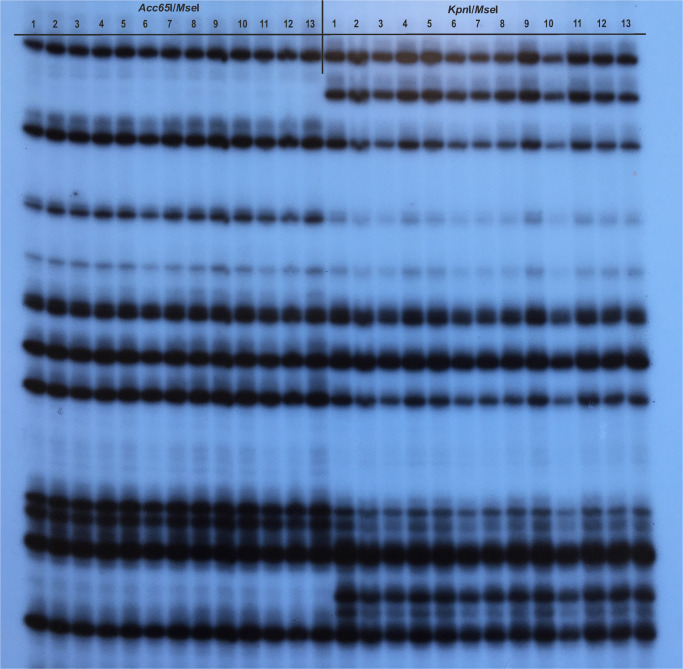
An example of the methylation-sensitive Amplified Fragment Length Polymorphism (metAFLP) profile generated with CXG-AGC and MCAC selective primers. The *Acc*65I/*Mse*I and *Kpn*I/*Mse*I reflected metAFLP platforms. Line 1 represents profiles of the donor, lines 2–7 and 8–13 profiles of regenerants obtained under M8 and M9 conditions, respectively.

In M1–M9 trials, a total of 335 *Acc*65I/*Mse*I and 326 *Kpn*I/*Mse*I shared markers were amplified using eight selective primer pairs. The metAFLP profiles (Figure 1) allowed the identification of four-digit banding patterns (events), with the prevailing “Z_1111” pattern reflecting a lack of change between the donor plant and its regenerants. The next most common events were “Z_1100” and “Z_0011,” which were assigned to the DNA methylation status of the donor and regenerant, respectively. No events corresponding to the “Z_0110” and “Z_1001” patterns (Supplementary Table S2) were detected.

Conversion of events into the metAFLP quantitative characteristics related to DNMV, DMV, and SV of the CHH, CHG, and CG sequence contexts showed that all characteristics exhibited some level of variation (Table 1). CHG_DNMV and CHG_DMV prevailed over CG_DMV and CG_DNMV. Moreover, DNA demethylation was lower than *de novo* methylation in both methylation contexts, and SV was the most abundant type of variation identified among embryo-derived regenerants. Among all trials, M7–M9 trials with the highest concentration of copper ions in the induction medium showed the highest mean values of SV in regenerants, ranging from 3.37 to 3.83%, whereas in other trials, SV values mostly approximated to 2.0%.

The results of one-way ANOVA were only significant for CHG_DMV [*F*(*8*) = 1009815.8, *p* < 0.0001, *R*^2^*_*a*__*dj*_* = 1], CHG_DNMV [*F*(*8*) = 428448.3, *p* < 0.0001, *R^2^_*a*__*dj*_* = 1], and SV [*F*(*8*) = *14.6, p* < 0.0001, *R*^2^*_*a*__*dj*_* = *0.762*] metAFLP characteristics, thus demonstrating mean differences between trials. Tukey’s test revealed significant differences in CHG_DMV between three groups of trials: group A (M1, M2, M3), group B (M4, M5, M6, M7), and group C (M8, M9). Similarly, CHG_DNMV showed significant differences between two groups of trials: group A (M1, M2, M3, M4, M5, M6, M7) and group B (M8, M9). SV showed less variability among trials; differences in SV were observed among four partially overlapping groups of trials: group A(M7, M8, M9), B (M2, M8, M9), C (M2, M6, M9), and D (M1, M2, M3, M4, M5, M9).

Pearson correlation analysis showed that SV was significantly correlated with Cu^2+^, CHG_DMV, and CHG_DNMV (Table 2). Furthermore, CHG_DMV and CHG_DNMV were correlated with Cu^2+^ but not with silver ion concentration. CHG_DNMV was correlated with the duration of incubation on the induction medium. Moreover, Cu^2+^ was correlated with SV. The correlation between CHG_DMV and CHG_DNMV was −0.62, while that between CG_DMV and CG_DNMV was 0.38.

**TABLE 2:** Pearson correlation analysis of tissue culture conditions and metAFLP characteristics.

Parameters^†^	[Cu^2+^]	[Ag^+^]	Time	CG_DMV	CHG_DMV	CG_DNMV	CHG_DNMV	SV
[Cu^2+^]	1	0.000	0.000	–0.022	0.866**	0.250*	0.656**	0.679**
[Ag^+^]	0.000	1	0.000	0.082	0.002	–0.039	0.136	–0.070
Time	0.000	0.000	1	0.120	–0.003	–0.248	−0.328*	0.015
CG_DMV	–0.022	0.082	0.120	1	–0.089	−0.622**	0.090	–0.078
CHG_DMV	0.866**	0.002	–0.003	–0.089	1	0.097	0.385**	0.372**
CG_DNMV	0.250*	–0.039	–0.248	−0.622**	0.097	1	0.393**	0.164
CHG_DNMV	0.656**	0.136	−0.328*	0.090	0.385**	0.393**	1	0.505**
SV	0.679**	–0.070	0.015	–0.078	0.372**	0.164	0.505**	1

^†^[Cu^2+^] and [Ag^+^], concentrations of copper and silver ions, respectively, in the induction medium; Time, duration of incubation on the induction medium; CG_DMV and CHG_DMV, DNA demethylation of the CG and CHG sequence contexts; CG_DNMV and CHG_DNMV, de novo DNA methylation of the CG and CHG sequence contexts; SV, sequence variation. Data represent Pearson’s correlation coefficients. Asterisks indicate significant correlation (*p < 0.05, **p < 0.01; one-tailed Student’s t-test).

Regression analysis showed that the model encompassing SV, CHG_DMV, and CHG_DNMV was significant [*F*(*2*) = 8.675, *p* = 0.001]. SV could be regressed on CHG_DMV and CHG_DNMV [*F_*chang*__*e*_*(*2, 42*) = 8.675, *p* = 0.001] with *R*^2^*_*adj*_* = 0.259. Unstandardized and standardized coefficients for CHG_DMV were 0.681 and 0.209, respectively, while those for CHG_DNMV were 1.55 and 0.425, respectively; however, only the last one (CHG_DNMV) was significant (*t* = 3.02, *p* = 0.004). The Shapiro–Wilk test (0.86, *p* = 0.000) and Kolmogorov–Smirnov test with Lilliefors significance correction (0.2, *p* < 0.01) violated the normality of the outcome (SV) variable. However, skewness (0.467, *SE* = 0.354) and kurtosis (−0.531, *SE* = 0.695) were not significantly affected. Visual inspection of the normal quantile–quantile (Q-Q) plot (data not shown) revealed almost normal distribution. Collinearity of the CHG_DNMV and CHG_DMV was not violated [Tolerance = 0.852, Variance inflation factor (VIF) = 1.174 for both variables]. Additionally, no outliers were detected based on Cook’s distance (0.2, *SD* = 0.03).

**FIGURE 2:**
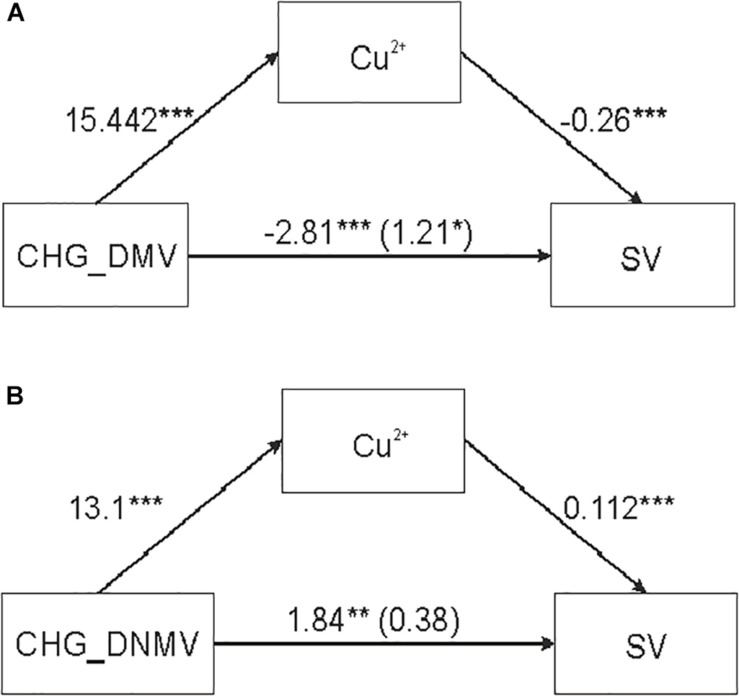
Simple mediation analysis illustrating the effect of copper ions on the relationship between sequence variation (SV) and DNA demethylation (DMV) and *de novo* DNA methylation (DNMV) in barley regenerants. **(A,B)** Relationship between CHG_DMV and SV **(A)** and between CHG_DNMV and SV **(B)** mediated by copper ions. *p < 0.05, **p < 0.01, ***p < 0.001.

A significant mediation effect of CHG_DMV and CHG_DNMV [independent variables (IDs)] on SV (outcome variable) through copper ions (mediator) (Table 3 and Figure 2) was evaluated based on significant indirect effects (IEs) of copper, as indicated by bootstrap values. The IEs were positive for the model with CHG_DMV and CHG_DNMV predictors. The significance of mediation was assessed by the Goodman test (CHG_DMV: *Z* = 6.43, *p* < 0.001; CHG_DNMV: *Z* = 3.36, *p* < 0.001). The variance accounted for (VAF) value, represented as the beta coefficient ratio of the IE to the total effect, and the strength of the mediation were 331.4 (CHG_DMV) and 79.3%, respectively, indicating full and partial mediations (Hair et al., 2011), respectively.

**TABLE 3:** Outcomes of mediation analyses illustrating relationships between CHG_DMV/CHG_DNMV and SV assessing IEs.

Model^†^	Effects§						95% CI^‡^	
	*R*^2^	*c’*	*a*	*b*	*c*	*IE*	*L*	*U*
CHG_DMV→Cu^2+^→SV	0.647	−2.808***	15.442***	0.26***	1.214*	4.021	3.01	5.12
CHG_DNMV→Cu^2+^→SV	0.467	0.382	13.103***	0.112***	1.844***	1.461	0.41	2.11
CG_DMV→Cu^2+^→SV	0.465	–7.941	–15.221	0.124**	–9.826	–1.885	–24.273	29.663
CG_DNMV→Cu^2+^→	0.461	–0.013	3.125	0.124**	0.376	0.389	–0.066	0.939
CHG_DMV→Ag^+^→SV	0.144	1.214**	0.185	0.002	1.214	–0.0004	–1.723	0.179
CHG_DNMV→Ag^+^→SV	0.275	1.914**	17.663	–0.004	1.844	–0.07	–0.345	0.164
CG_DMV→Ag^+^→SV	0.01	–9.166	367.55	–0.002	–9.826	–0.066	–11.234	5.214
CG_DNMV→Ag^+^→SV	0.03	0.37	–3.167	–0.002	0.376	0.006	–0.084	0.163

^†^CHG_DMV and CHG_DNMV, DNA demethylation and de novo DNA methylation, respectively, of the CHG sequence context (predictors); Cu^2+^, copper ion concentration (mediator); Ag^+^, silver ion concentration (mediator); SV, sequence variation (outcome variable).§ R^2^, amount of variance explained by the model; c’, direct effect of predictor (IDs) on the outcome (SV) while controlling for the mediator (Cu^2+^, Ag^+^); a, effect of the predictor (ID) on the mediator (Cu^2+^, Ag^+^); b, effect of the mediator (Cu^2+^, Ag^+^) on the outcome (SV); c, total effect focal predictor (CHG_DMV and CHG_DNMV, CG_DMV and CG_DNMV) on the outcome (SV); IE, indirect effect of the predictor (ID) on the outcome (SV) through the mediator. Asterisks indicate significant values (*p < 0.05, **p < 0.01; ***p < 0.001).^‡^CI, confidence interval; L and U, lower and upper limits, respectively, of 95% CI.

No mediation was evaluated for copper ion concentration when CG_DMV and CG_DNMV were used as predictors. Additionally, no mediation effect of silver ions was detected in the relationship between any of the predictors (CG_DMV, CG_DNMV, CHG_DMV, and CHG_DNMV) (data not shown) (Table 3).

## Discussion

To determine the quantitative SV and DNA methylation change characteristics of regenerants using the metAFLP approach, obtaining tissue culture explant from a DH genotype after the generative cycle is a prerequisite (Bednarek et al., 2007; Machczyńska et al., 2014b; Orłowska and Bednarek, 2020; Orłowska et al., 2020). It is also crucial to have regenerants representing all trials from a single donor plant; otherwise, metAFLP quantitative characteristics might be disturbed by putative variation of donor plants [even if they originated from the same progenitor (Bednarek et al., 2007; Machczyńska et al., 2015)] due to the so-called genotype effect (Anu et al., 2004; Flinn et al., 2020). Having this in mind, only one experimental set included regenerants representing all trials with a minimum of five regenerants per trial. The other sets were underrepresented in different trials and made repeated experiments impossible (Supplementary Figure S1). The immature zygotic embryo culture approach allowed the regeneration of a sufficient number of plants equally represented in all experimental trials differing in induction medium composition and tissue culture duration. The regenerants were morphologically identical to the donor. This was unexpected since copper nanoparticles (to a lesser extent than in the presence of zinc nanoparticles) have been shown to affect chlorophyll-a in *Pistia stratiotes* L. (Araceae), leading to morphological variation (Olkhovych et al., 2016). It should be noted that copper and zinc function together to induce oxidative stress (Lee et al., 2002). Thus, the lack of morphological variation among embryo-derived regenerants was probably due to the constant concentration of zinc ions in the induction medium. However, the absence of morphological variation does not necessarily imply that changes in DNA sequence and DNA methylation pattern related to tissue culture conditions were not induced.

The metAFLP analysis revealed changes in SV as well as in the DNA demethylation and *de novo* methylation of distinct sequence contexts; this is consistent with the results obtained using anther culture-derived barley regenerants (Bednarek and Orłowska, 2020a). It should be stressed that CHH-related variation was not evaluated. This is because methylation of an asymmetric context is much less than that of symmetric contexts (Cokus et al., 2008). A higher level of differences between trials was caused by the comparison of CHG_DNMV and CHG_DMV with CG_DMV and CG_DNMV, which is surprising since CG methylation is presumably more abundant in plants than CHG methylation (Cokus et al., 2008). The identification of increased variation within the CHG context might reflect the as-yet-unrecognized aspects of embryogenesis rather than metAFLP approach preferences. Finally, the amount of SV was either equal to or slightly greater than the total DMV and DNMV, which is not fully congruent with our previous studies on barley regenerants (Bednarek et al., 2007), where DNA methylation pattern changes exceeded those related to SV by at least two-fold. This difference in SV between the two studies could be attributed to varying copper ion concentrations used in the present study. Furthermore, increased SV in trials with the highest copper ion concentrations is consistent with studies on humans, thus documenting the role of copper ions in inducing mutations that affect the methylated cytosine residue within CG sequences (Lee et al., 2002). In the current study, the CHG contexts (specific to plants) were preferentially affected by SV compared with the CG context.

The results of ANOVA indicated that changes in CHG_DMV, CHG_DNMV, and SV could be explained by experimental trials. However, ANOVA failed to explain the variance between trials based on changes in CG_DMV and CG_DNMV. Thus, methylation changes affecting CHG contexts might be typical for embryo-derived regenerants. The reason why ANOVA results were insignificant for CG_DMV and CG_DNMV may be related to embryogenesis as CG methylation could be reestablished based on the DNA methylation pattern of chromosome homologs, whereas methylation of the CHG sequences may partly depend on epigenetic mechanisms (Saze et al., 2012; Niederhuth and Schmitz, 2014; Thiebaut et al., 2019; Liu and Lang, 2020), which are less precise than methylation mechanisms. This is supported by the fact that methylation changes affecting CG contexts were lower than those related to the CHG context.

In this study, SV showed a significant correlation with CHG_DMV and CHG_DNMV, suggesting that DNA demethylation and *de novo* DNA methylation may explain DNA sequence variation. Linear regression of SV on CHG_DNMV (but not on CHG_DMV) indicates that *de novo* methylation of the symmetric CHG sequence context plays a vital role in the generation of SV. The lack of the identified CG sequence context may be attributed to the reestablishment of methylation patterns during cell division *via* replication mechanisms and the avoidance of epigenetic modifications during the process. By contrast, the contribution of CHG context in DNMV may indicate the participation of epigenetic pathways in the reestablishment of methylation patterns. This notion seems to be confirmed by our studies of anther culture-derived barley regenerants (Orłowska and Bednarek, 2020), where the role of both CG and CHG contexts was demonstrated. The necessity of chromosome doubling in the case of anther culture-derived regenerants might explain the difference in DNA methylation context variation between the two types of plant regeneration methods. The results of this study are congruent with studies on *Arabidopsis thaliana*, indicating that methylated cytosines could be a source of mutations in the DNA sequence (Kiselev et al., 2019). It should be stressed, however, that linear regression analysis violated the assumption of normal distribution. Since normal distribution is assumed to be a prerequisite for linear regression, the results of this study should be treated with caution.

Both CHG_DMV and CHG_DNMV were correlated with Cu^2+^, and Cu^2+^ was correlated with SV. Moreover, CHG_DMV and CHG_DNMV were also correlated with SV, indicating that mediation analysis between CHG_DMV and CHG_DNMV with SV, in the presence of Cu^2+^ (but not Ag^+^) as a mediator, is well grounded. Mediation analysis indicated that copper ions act as a mediator between CHG_DMV/_DNMV and SV during embryogenesis. The observed relationship is not surprising because copper influences the mitochondrial respiratory chain (Garcia et al., 2014), specifically complex IV (COX), as shown in humans (Borghouts et al., 2002), thus affecting ATP production. ATP is needed for the biosynthesis of SAM from methionine by SAM synthetase (Taylor et al., 2009). Furthermore, SAM, the product of the Yang cycle, is required for DNA methylation (Roje, 2006), which may lead to DNA SV in the presence of copper ions (Lee et al., 2002). The role of copper, as a mediator, in inducing SV from CG and CHG contexts has also been reported in barley anther culture (Bednarek and Orłowska, 2020a). Interestingly, however, in anther culture, SV was also related to CG_DMV, whereas in embryo-derived regenerants, only the role of the CHG context was revealed. The observed difference could be explained by the precise control over *de novo* methylation and demethylation of the CHG sequence context during embryogenesis compared with androgenesis and possibly the involvement of different pathways affecting DNA methylation changes during both processes. Our results are consistent with those of previous studies (Yruela, 2009; Printz et al., 2016), which showed that copper ions play a crucial role in plant cell function and participate in a complex network of interactions that influence the SV of CHG contexts in plants. Assuming that CHG_DNMV and CHG_DMV explain only a small fraction of SV shared among embryo-derived regenerants, as indicated by ANOVA, factors other than copper ions, such as mobile elements (Smith et al., 2012; Orłowska et al., 2016; Masuta et al., 2017), are involved in SV during plant tissue culture. Evidently, however, a higher concentration of copper ions in the induction medium led to a higher level of SV. This result is in agreement with data demonstrating copper toxicity in plants (Nawrot-Chorabik, 2017).

To our surprise, silver ions failed to exhibit any significant mediation between any DNA *de novo* methylation/demethylation and sequence contexts; this is not congruent with the results of anther culture (Orłowska and Bednarek, 2020) possibly because the cell wall of zygotic embryos limits the accessibility of ions in the cell, as suggested by Printz et al. (2016) for copper. This suggests that silver ions are less critical for zygotic embryo culture than for anther culture. Interestingly, the duration of barley zygotic embryo culture was not correlated with Cu^2+^ and SV. The result is in opposition to that of barley anther culture (Orłowska and Bednarek, 2020). The statistical insignificance of the duration of culture was probably caused by the presence of homologous chromosomes, which could facilitate the repair of putative SV. However, further investigation is needed to test this possibility. Nonetheless, SV induction during embryogenesis is certainly controlled differently than that during androgenesis. Alternatively, the differences in the mediations between anther culture and embryo culture, as shown previously, might be due to the way the two explant types have to switch their fate to an embryogenic one.

The putative limitation of this study is the sample size (number of regenerants). There is no simple formula that can be used to determine the minimum sample size for mediation analysis (Pan et al., 2018). Bootstrapping approach was suggested as a method of choice for sample sizes ranging from 20 to 80 (Shrout and Bolger, 2002; Preacher and Hayes, 2004). However, in some cases, mediation analysis with bootstrapping may overestimate effects (Koopman et al., 2015). It was demonstrated however, that under some conditions, mediation analysis can be successfully performed on a sample size as small as 22 (Pan et al., 2018). Thus, our sample size of 45 regenerants fulfills the requirement for mediation analysis. Furthermore, mediation analysis successfully identified significant relationships between CHG_DMV/CHG_DNMV and SV mediated by copper ions. The significance of the models was confirmed by the Goodman test and bootstrapping of IEs. Assuming that our data are congruent with the biological role of copper ions in biochemical pathways, we believe that the mediation analysis results presented in this study reflect the real biological phenomenon. However, it would be of value to investigate a larger sample size to confirm our results.

## Conclusion

The present study demonstrates that different sequence methylation contexts affect SV in somatic embryo-derived regenerants because of the presence of copper ions in the induction medium. Copper ions may mediate SV, affecting the mitochondrial respiratory chain, L-methionine cycle, and mutation-prone CHG DNA contexts. In embryo-derived regenerants, the CHG contexts are responsible for SV, when copper ions act as a mediator. The lack of a mediating effect of silver ions during embryo culture was surprising. We speculate that the cell wall of zygotic embryos limits the accessibility of silver ions in the cell, and thus silver ions do not compete for cellular complexes involved in the respiratory chain. Zygotic embryo cultures may differ in TCIV at the DNA sequence and methylation levels, suggesting that slightly different mechanisms or pathways are involved during plant regeneration *via* anther and embryo culture. Studies dedicated to evaluating models that describe relationships between biochemical pathways and TCIV aim to develop practical tools for *in vitro* tissue culture manipulations, thus enhancing the understanding of and allowing control over parameters affecting different aspects of SV. Such models would be of value from both scientific and practical points of view.

## Data Availability Statement

The original contributions presented in the study are included in the article/Supplementary Materials, further inquiries can be directed to the corresponding author/s.

## Author Contributions

RO and PB conceived the project, designed the research, analyzed the data, and wrote the first draft of the manuscript. RO performed the experiments. RO and JZ prepared plant material. RO, PB, and JZ revised and finalized the manuscript. All authors contributed to the article and approved the submitted version.

## Conflict of Interest

The authors declare that the research was conducted in the absence of any commercial or financial relationships that could be construed as a potential conflict of interest.

## Footnotes

1 http://quantpsy.org/sobel/sobel.htm

## Supplementary Material

Supplementary Figure S1 Experimental design. Starting materials was represented by individual plants of the NAD2 barley line that were subjected to androgenesis (anther culture) to obtain DH regenerants. After a single round of generative cycle the progeny of a single regenerant encompassing twenty-four plants was used as a source of explants (immature zygotic embryos). The immature embryos of the given donor plant were subjected toward nine experimental conditions (M1-M9 trials) and formed a set. Only a single set resulted in regenerants that were regenerated under all trial conditions and was represented by at least five regenerants. In order to have equal representation of all regenerants in all trials five regenerants were used for further molecular (metAFLP) procedures.

Supplementary Figure S2 Donor plants of barley.

## Abbreviations

2,4-D: 2,4-dichlorophenoxyacetic acid
ATP: adenosine triphosphate
BAP: 6-benzylaminopurine
BER: base excision repair
DArTseqMet: Diversity Arrays Technology Sequencing Methylation Analysis
DH: doubled haploid
DMV: demethylation
DNMV: *de novo* methylation
metAFLP: methylation-sensitive Amplified Fragment Length Polymorphism
MethylRad: DNA methylation profiling method
MMR: mismatch repair
MSAP: methylation-sensitive amplification polymorphism
NAA: 6-naphthalene acetic acid
NER: nucleotide excision repair
PacBio: Pacific BioScience
ROS: reactive oxygen species
SAM: S-adenosyl -L-methionine
SV: sequence variation
TCIV: tissue culture-induced variation.
