# Supplementary material for: Copper Ions Induce DNA Sequence Variation in Zygotic Embryo Culture-Derived Barley Regenerants
Source: Front Plant Sci. 2021 Feb 4;11:614837. doi: 10.3389/fpls.2020.614837 (PMC7889974; doi:10.3389/fpls.2020.614837)
Supplement: Supplementary Figure S1 — Experimental design. Starting materials was represented by individual plants of the NAD2 barley line that were subjected to androgenesis (anther culture) to obtain DH regenerants. After a single round of generative cycle the progeny of a single regenerant encompassing twenty-four plants was used as a source of explants (immature zygotic embryos). The immature embryos of the given donor plant were subjected toward nine experimental conditions (M1-M9 trials) and formed a set. Only a single set resulted in regenerants that were regenerated under all trial conditions and was represented by at least five regenerants. In order to have equal representation of all regenerants in all trials five regenerants were used for further molecular (metAFLP) procedures. [file Data_Sheet_1.PDF]

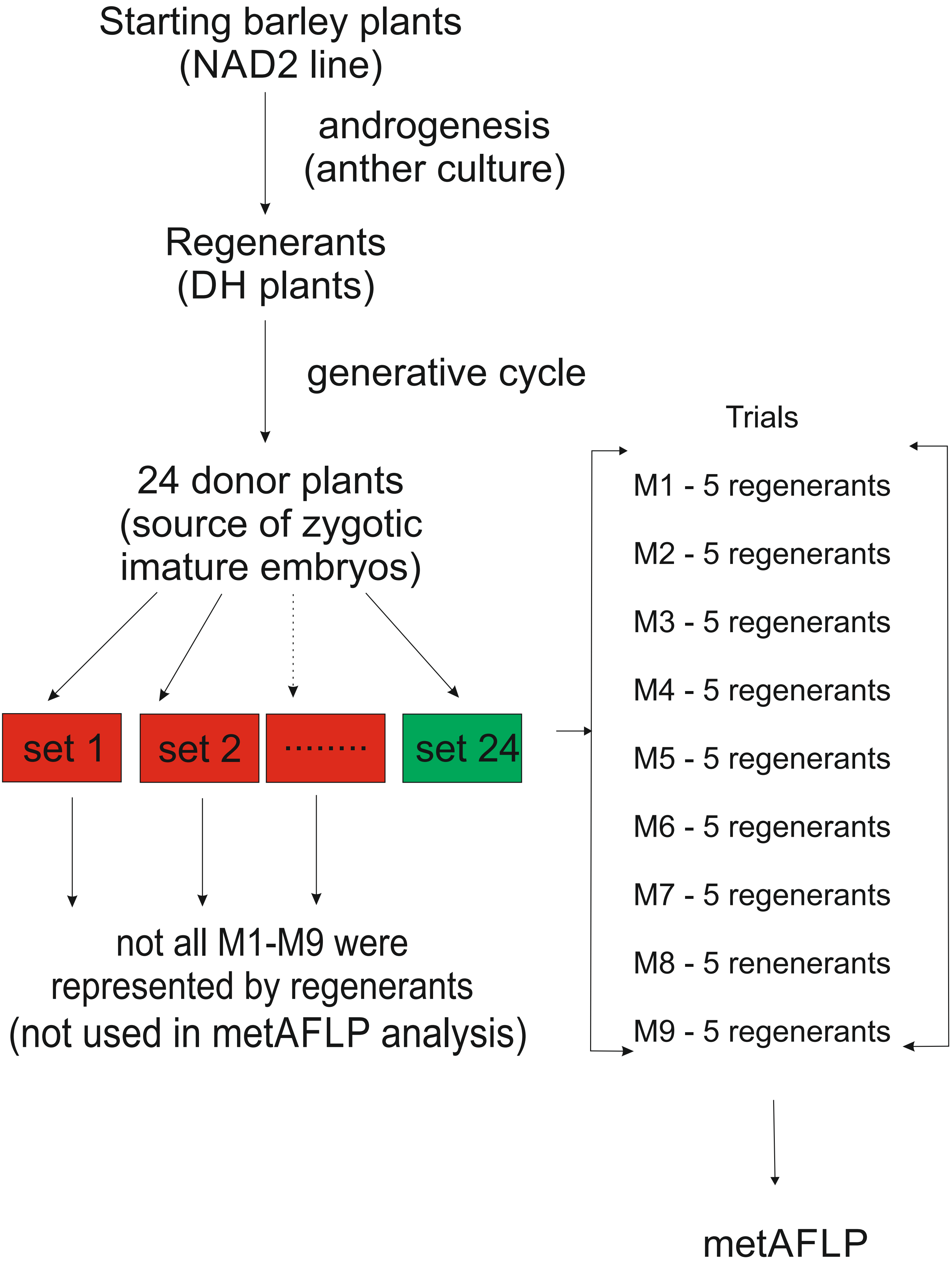

Starting barley plants
(NAD2 line)
androgenesis
(anther culture)
Regenerants
(DH plants)
generative cycle
24 donor plants
(source of zygotic
imature embryos)
set 1
set 2
....
set 24
not all M1-M9 were
represented by regenerants
(not used in metAFLP analysis)
Trials
M1 - 5 regenerants
M2 - 5 regenerants
M3 - 5 regenerants
M4 - 5 regenerants
M5 - 5 regenerants
M6 - 5 regenerants
M7 - 5 regenerants
M8 - 5 renenerants
M9 - 5 regenerants
metAFLP
