# Supplementary figures and images for: Copper Ions Induce DNA Sequence Variation in Zygotic Embryo Culture-Derived Barley Regenerants
Source: Front Plant Sci. 2021 Feb 4;11:614837. doi: 10.3389/fpls.2020.614837 (PMC7889974; doi:10.3389/fpls.2020.614837)

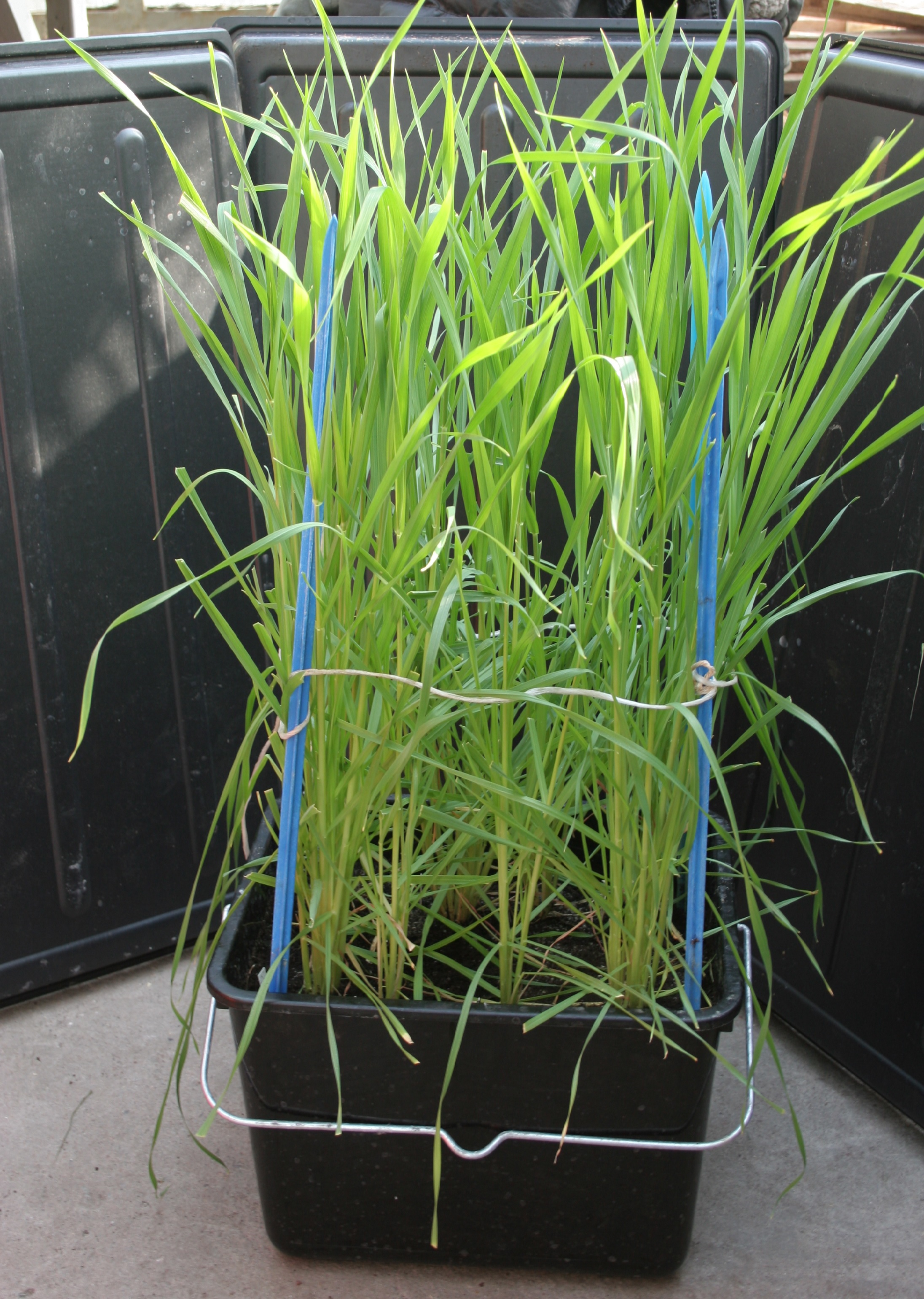

Supplement: Supplementary Figure S2 — Donor plants of barley. [file Image_2.JPEG]
