# Supplementary material for: Copper Ions Induce DNA Sequence Variation in Zygotic Embryo Culture-Derived Barley Regenerants
Source: Front Plant Sci. 2021 Feb 4;11:614837. doi: 10.3389/fpls.2020.614837 (PMC7889974; doi:10.3389/fpls.2020.614837)
Supplement: Supplementary file 3 [file Table_1.DOCX]

**SUPPLEMENTARY MATERIAL**

**TABLE S1** Oligonucleotides applied for metAFLP in barley studies.

| metAFLP oligomer | Sequence 5’→3’ |
| --- | --- |
| Labeled ^32^P selective oligonucleotides |  |
| CG-GAC | CA TGC GTA CAG TAC CGA C |
| CG-GGC | CA TGC GTA CAG TAC CGG C |
| CXG-AGA | CA TGC GTA CAG TAC CAG A |
| CXG-AGC | CA TGC GTA CAG TAC CAG C |
| CXG-AGG | CA TGC GTA CAG TAC CAG G |
| CXG-TTG | CA TGC GTA CAG TAC CTT G |
| CXX-ATT | CA TGC GTA CAG TAC CAT T |
| CXX-TAA | CA TGC GTA CAG TAC CTA A |
| Selective oligonucleotides |  |
| M-CAC | GAT GAG TCC TGA GTA ACA C |
| M-CGT | GAT GAG TCC TGA GTA ACG T |
| M-CTA | GAT GAG TCC TGA GTA ACT A |
| Primer combination used in analysis | |
| CG-GAC/MCGT | |
| CG-GGC/MCAC | |
| CXG-AGA/MCGT | |
| CXG-AGC/MCAC | |
| CXG-AGG/MCGT | |
| CXG-TTG/MCGT | |
| CXX-ATT/MCAC | |
| CXX-TAA/MCGT | |

**TABLE S2** The events evaluated for the M1-M9 trials.

| Events | Trials | | | | | | | | |
| --- | --- | --- | --- | --- | --- | --- | --- | --- | --- |
|  | M1 | M2 | M3 | M4 | M5 | M6 | M7 | M8 | M9 |
| Z_0000 | 12 | 10 | 10 | 13 | 15 | 19 | 14 | 12 | 12 |
| Z_0001 | 0 | 1 | 0 | 1 | 1 | 0 | 0 | 0 | 0 |
| Z_0010 | 1 | 4 | 0 | 0 | 0 | 7 | 9 | 10 | 10 |
| Z_0011 | 64 | 61 | 65 | 60 | 60 | 53 | 51 | 50 | 50 |
| Z_0100 | 13 | 13 | 14 | 11 | 9 | 6 | 10 | 12 | 13 |
| Z_0101 | 0 | 1 | 1 | 0 | 0 | 0 | 1 | 1 | 0 |
| Z_0110 | 0 | 0 | 0 | 0 | 0 | 0 | 0 | 0 | 0 |
| Z_0111 | 20 | 20 | 20 | 25 | 25 | 25 | 25 | 25 | 25 |
| Z_1000 | 10 | 10 | 10 | 10 | 10 | 10 | 10 | 15 | 15 |
| Z_1001 | 0 | 0 | 0 | 0 | 0 | 0 | 0 | 0 | 0 |
| Z_1010 | 0 | 0 | 0 | 0 | 0 | 0 | 0 | 5 | 5 |
| Z_1011 | 17 | 14 | 16 | 14 | 14 | 17 | 16 | 23 | 24 |
| Z_1100 | 70 | 67 | 68 | 69 | 69 | 70 | 68 | 65 | 65 |
| Z_1101 | 15 | 18 | 17 | 16 | 16 | 15 | 17 | 15 | 15 |
| Z_1110 | 5 | 5 | 5 | 5 | 5 | 5 | 13 | 5 | 5 |
| Z_1111 | 733 | 736 | 734 | 736 | 736 | 733 | 726 | 722 | 721 |
